# Germline Mutation in *MUS81* Resulting in Impaired Protein Stability is Associated with Familial Breast and Thyroid Cancer

**DOI:** 10.3390/cancers12051289

**Published:** 2020-05-20

**Authors:** Maisa Pinheiro, Fernanda Cristina Sulla Lupinacci, Karina Miranda Santiago, Sandra Aparecida Drigo, Fabio Albuquerque Marchi, Carlos Eduardo Fonseca-Alves, Sonia Cristina da Silva Andrade, Mads Malik Aagaard, Tatiane Ramos Basso, Mariana Bisarro dos Reis, Rolando André Rios Villacis, Martin Roffé, Glaucia Noeli Maroso Hajj, Igor Jurisica, Luiz Paulo Kowalski, Maria Isabel Achatz, Silvia Regina Rogatto

**Affiliations:** 1 Faculty of Medicine, Sao Paulo State University, UNESP, Botucatu SP 18618-687, Brazil; maisapinheiro12@gmail.com; 2 International Research Center, A.C. Camargo Cancer Center, São Paulo SP 01508-010, Brazil; flupinacci@accamargo.org.br (F.C.S.L.); karinamirsant@gmail.com (K.M.S.); biomarchi@gmail.com (F.A.M.); tatianebasso2015@gmail.com (T.R.B.); marianabisarro@yahoo.com.br (M.B.d.R.); mroffe@accamargo.org.br (M.R.); ghajj@cipe.accamargo.org.br (G.N.M.H.); lpkowalski@accamargo.org.br (L.P.K.); 3 Department of Surgery and Orthopedics, Experimental Research Unity, Faculty of Medicine, São Paulo State University, UNESP, Botucatu SP 18618-687, Brazil; sandradrigo@gmail.com; 4 Department of Veterinary Surgery and Anesthesiology, São Paulo State University, UNESP, Botucatu SP 18618-681, Brazil; carlos.e.alves@unesp.br; 5 Department of Genetics and Evolutionary Biology, University of São Paulo, USP, São Paulo SP 05508-090, Brazil; soniacsandrade@ib.usp.br; 6 Department of Clinical Genetics, Vejle University Hospital, 7100 Vejle, Denmark; mads.jorgensen@rsyd.dk; 7 Department of Genetics and Morphology, Institute of Biological Sciences, University of Brasília, UnB, Brasília DF 70910-900, Brazil; rolando.andre@unb.br; 8 Krembil Research Institute, UHN, University of Toronto, Toronto, ON M5G 2C4, Canada; juris@ai.utoronto.ca; 9 Institute of Neuroimmunology, Slovak Academy of Sciences, 845 10 Bratislava, Slovakia; 10 Cancer Genetics Unit, Centro de Oncologia, Hospital Sirio Libanês, São Paulo SP 01308-050, Brazil; miachatz@gmail.com; 11 Department of Clinical Genetics, Vejle University Hospital, Institute of Regional Health Research, University of Southern Denmark, 5000 Odense, Denmark

**Keywords:** exome sequencing, *MUS81*, breast cancer, thyroid cancer, functional analysis, familial cancer

## Abstract

Multiple primary thyroid cancer (TC) and breast cancer (BC) are commonly diagnosed, and the lifetime risk for these cancers is increased in patients with a positive family history of both TC and BC. Although this phenotype is partially explained by *TP53* or *PTEN* mutations, a significant number of patients are negative for these alterations. We judiciously recruited patients diagnosed with BC and/or TC having a family history of these tumors and assessed their whole-exome sequencing. After variant prioritization, we selected *MUS81* c.1292G>A (p.R431H) for further investigation. This variant was genotyped in a healthy population and sporadic BC/TC tissues and investigated at the protein level and cellular models. *MUS81* c.1292G>A was the most frequent variant (25%) and the strongest candidate due to its function of double-strand break repair. This variant was confirmed in four relatives from two families. MUS81 p.R431H protein exhibited lower expression levels in tumors from patients positive for the germline variant, compared with wild-type BC, and normal breast and thyroid tissues. Using cell line models, we showed that c.1292G>A induced protein instability and affected DNA damage response. We suggest that *MUS81* is a novel candidate involved in familial BC/TC based on its low frequency in healthy individuals and proven effect in protein stability.

## 1. Introduction

Thyroid cancer (TC) is the most common secondary tumor in patients diagnosed with primary breast cancer (BC) [1,2]. Similarly, BC is reported as the most frequent second primary tumor in TC patients [3,4,5]. The lifetime risk for these cancers is increased in patients with a positive family history of both TC and BC [3,6,7], and clinical cancer surveillance might be appropriate for some of the cases [8]. In a large cohort of 13,798 BC Chinese patients, family history of cancer was the only predictor of secondary TC in BC patients [1]. Studies to investigate mechanisms involved in this association are necessary [9].

Hereditary BC and TC are mainly related to Cowden syndrome in which 30% to 35% of patients are positive for *PTEN* pathogenic variants [10,11]. Breast cancer is the most frequent cancer in the Li–Fraumeni syndrome tumor spectrum, which is associated with pathogenic variants in *TP53* [12]. However, TC is also rarely described in Li–Fraumeni patients [13]. In addition to *PTEN* and *TP53*, other candidates were reported as presenting potential predisposition genes associated with familial BC/TC [14,15]. In Cowden or Cowden-like syndromes, *SDHx* and *KLLN* genes were reported as modifiers of the phenotype [16,17]. Two variants mapped in *PARP4* (p.G496V and p.T1170I) were detected among 14 unrelated individuals diagnosed with both BC and TC [14]. Four Polish founder variants in *CHEK2* (1100delC, IVS2+1G>A, del5395, and I157T) were described in TC patients who were also diagnosed with BC or had familial breast cancer history [15].

An association between BC and TC was also described in TC patients treated with surgery and exposed to radioiodine therapy. These patients presented a higher risk of developing a second primary cancer of the breast [18]. A plausible explanation is a deregulation of thyroid hormones (in TC and in other thyroid dysfunctions such as hyperthyroidism and hypothyroidism), which may have pro- and anti-oncogenic properties able to trigger BC development [19]. A recent study based on United States survivors (2000–2015) showed an increased risk of second primary papillary TC for several cancer types, including BC. According to these authors, the risk of developing papillary TC was not clearly associated with the treatment of the first tumor and shared risk factors could explain this association [20]. High-penetrance genes or genetic variants associated with this phenotype are poorly explored, and markers for preventive screening would benefit high-risk patients. Herein, the germline DNA of patients diagnosed with BC and/or TC and familial history of these tumors was whole-exome sequenced to investigate genetic variants potentially associated with hereditary BC and TC.

## 2. Results

### 2.1. Variant Identification and Prioritization

After applying stringent selection criteria, we selected 20 patients, out of 130, with personal and familial history of TC and BC (Table S1). DNA from peripheral blood samples was evaluated by whole-exome sequencing in 20 index patients from independent families and in three relatives from two families (F1: W6-1 and W6-2 and F2: W7-1). Common variants shared by F1 (W6, W6-1, W6-2) or F2 (W7, W7-1) family members were kept for that specific family, and the resulting variants were compared to those detected in the remaining 18 unrelated individuals of our cohort. The summary of variant prioritization is presented in Figure S1.

**Figure 1:**
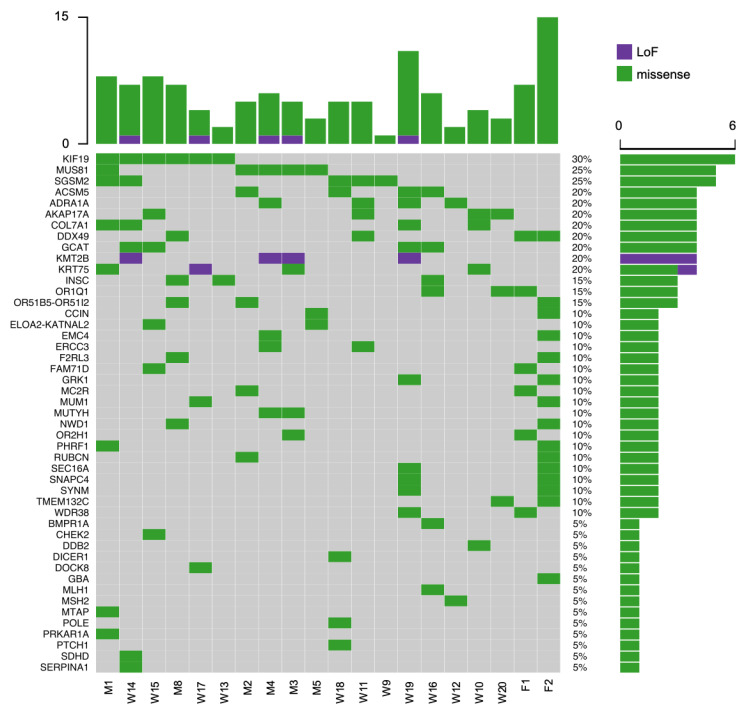
Schematic summary of genes after variant prioritization, including: 17 cancer-related genes with variants, genes with variants carried by families F1 or F2 and by an additional unrelated patient, recurrently altered genes, and genes with recurrent variants. Top bar plot: number of variants by patient/family; Right bar plot: number of variants by gene.

We found 20 missense variants in 17 cancer-related genes [21,22] (Figure 1, Table S2). According to ClinVar [23], two variants were classified as pathogenic/likely pathogenic, including *MUTYH* c.1187G>A (detected in two index patients: M3 and M4), and *SERPINA1* c.1096G>A variant (patient W14). Five variants were classified as a conflicting interpretation of pathogenicity (*GBA* c.1223C>T, *SDHD* c.149A>G, *PRKAR1A* c.221G>A, *CHEK2* c.478A>G, *PTCH1* c.3947A>G) according to ClinVar (Table S2), each present in one family/patient (F2, W14, M1, W15, W18, respectively). Five variants were classified as uncertain significance (VUS) according to ClinVar (*POLE* c.6775C>T, *DICER1* c.3553G>A, *MSH2* c.80C>T, *MLH1* c.1852A>G, *COL7A1* c.802C>T), each present in one patient (W18 had both *POLE* c.6775C>T and *DICER1* c.3553G>A, W12, W16, W19, respectively). Eight variants had no classification in ClinVar but were classified as benign or VUS by the ACMG (American College of Medical Genetics)-compliant classifications (Ingenuity and InterVar). From these, *COL7A1* had two variants, c.7313C>G (patients M1 and W14) and c.3532C>T (patient W10). *ERCC3* had two variants, c.2077C>T and c.514C>T, detected in patients M4 and W11, respectively. Detailed information of all variants is described in Tables S2 and S3.

We expanded our analysis to other genes and focused on recurrently altered genes and/or recurrent variants found in more than one index case (Tables S2 and S3).

Twenty-one variants mapped in 19 genes were detected in all individuals from F1 or F2 and were also carried by an additional unrelated index patient (Figure 1, detailed in Table S2). We found seven variants in six genes (*OR1Q1, FAM71D, MC2R, DDX49, OR2H1, WDR38*) shared by all individuals of F1, and 14 variants in 15 genes (*OR51B5/OR51I2, PHRF1, TMEM132C, GRK1, EMC4, SYNM, MUM1, NWD1, F2RL3, DDX49, RUBCN, SNAPC4, SEC16A, CCIN*) shared by members of family F2. Two different variants mapped to *DDX49* were detected in F1 (c.61G>A) and F2 (c.665G>A).

We flagged genes that were ranked according to their mutation burden previously reported in other exome/whole-genome studies due to large gene size [24] (Table S3). From 566 genes that had at least two variants, 196 were among the 1000 most mutated genes in exome/whole-genome studies [24], including the top ones *TTN*, *OBSCN*, *LAMA5, MYH13* (Table S3). Other recurrently altered genes were *KIF19* (six variants), *SGSM2* (three variants and one variant in two patients), and *ACSM5, ELOA2/KATNAL2, INSC, KRT75* (with four variants each) (Figure 1, detailed in Table S3). These variants were distinctly distributed among patients, most of them detected in only one patient, except for *SGSM2* (one variant in two patients). Recurrent variants, found in four or more individuals, were mapped to five different genes, including *MUS81, ADRA1A, GCAT, AKAP17A,* and *KMT2B* (this last one is flagged as highly mutated, detailed in Table S3) (Figure 1) [24].

Considering the top most recurrent variants, *MUS81* c.1292G>A was the strongest candidate for further validation due to its high frequency (five of 20 patients: M1, M2, M3, M4, M5) and also for the gene function of double-strand break repair [25,26]. The heterozygous nonsynonymous missense variant *MUS81* c.1292G>A at codon 431 results in an arginine-to-histidine change (p.R431H). Three of five carriers were diagnosed with BC at 45 (M2), 49 (M4), and 39 (M5) years old, and the other two positive cases were diagnosed with papillary TC at age 53 (M1) and 40 (M3) years (Table S1). The germline variant was confirmed by Sanger sequencing (Figure S2). We also sequenced all exons of *MUS81* from the tumor DNA of patient M1, available from fresh frozen tissue, to investigate other variants in the TC tissue sample. We confirmed the presence of c.1292G>A and another germline variant c.344C>T (Figure S3). The variant c.344C>T has minor allele frequency (MAF) = 0.015 in Gnomad, being more common in the African population (MAF = 0.054), and its CADD (Combined Annotation Dependent Depletion) score is 17 (the CADD pathogenicity score threshold adopted in this study was > 20). Another *MUS81* missense variant, c.1168A>C, was detected in patient M4, predicted as pathogenic in silico (CADD score 26), and it was also confirmed by Sanger sequencing.

Three sisters (M2-1, M2-2, and M2-3) from index patient M2 and a brother (M5-1) from index patient M5 were tested for *MUS81* c.1292G>A variant using Sanger sequencing (Figure S4). These relatives had papillary TC or thyroid nodules with prophylactic thyroidectomy. All individuals were positive for the *MUS81* c.1292G>A variant.

Four of five patients positive for *MUS81* c.1292G>A had at least one other variant in a cancer-related gene (Table S2). One of these variants, *MUTYH* c.1187G>A (p.G396D) identified in heterozygous in patients M3 and M4, was predicted as pathogenic in ClinVar. Other cancer-related genes, such as *COL7A1*, *PRKAR1A*, *ERCC3,* and *MTAP*, had variants with a conflicting interpretation of pathogenicity or VUS, or they were not reported in ClinVar (Table S2).

**Figure 2:**
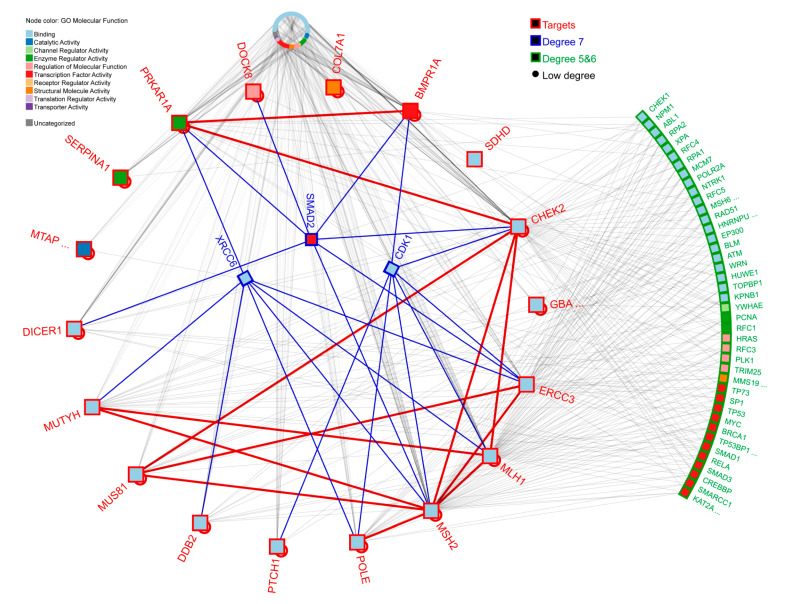
Protein–protein interaction network of 17 cancer related genes and *MUS81*, highlighting the most likely biologically relevant links among the selected proteins. Nodes represent proteins and lines represent physical protein interactions between them. Red outline identifies the 18 targets, including *MUS81* and 17 cancer-related genes, with variants identified by whole-exome sequencing. Gray lines represent low connectivity (low degree), while blue lines link proteins with the highest degree between target genes via *XRRC6*, *SMAD2* and *CDK1*, and red lines represent direct interactions between targets.

We constructed a protein–protein interaction (PPI) network with *MUS81* and 17 cancer-related genes detected with a variant in our exome analysis, to identify biological relationships among them. From the 17 cancer-related related genes, three (*CHEK2, ERCC3*, and *MSH2*) directly interact with *MUS81* and four (*PRKAR1A*, *MLH1*, *MUTYH*, *POLE*) indirectly interact with *MUS81* via *CHEK2*, *ERCC3*, and *MSH2* (Figure 2). Nine genes showed direct interaction among each other. Three non-target proteins (*XRCC6*, *SMAD2*, and *CDK1*) showed high connectivity to the 18 genes used in this analysis. Other non-target proteins that had at least one direct physical interaction with one of the 18 targets were retrieved from the Integrated Interactions Database (IID) [27]. We also performed an enrichment pathway analysis using the 18 PPI targets with PathDIP software [28]. Seventy-nine pathways were significantly enriched after Bonferroni correction for multiple comparisons (*p*-value < 0.05) (Table S4; significant *p*-values are in bold). Interestingly, *MUS81*, *CHEK2*, *ERCC3*, *MSH2*, *PRKAR1A*, *MLH1*, *MUTYH*, and *POLE* were simultaneously represented in 41 enriched pathways, with the top significant ones being Panther Pathway: DNA replication (*p* = 5.7 × 10^−6^), KEGG: nucleotide excision repair (*p* = 1.3 × 10^−5^), and Reactome: lagging strand synthesis (*p* = 1.8 × 10^−5^).

### 2.2. MUS81 c.1292G>A Genotyping

DNA target genotyping was assayed in peripheral blood samples from healthy individuals with no family cancer history and in fresh frozen tumor samples from sporadic BC and TC (Figure S5). We did not have blood samples from patients with sporadic TC and BC. Allelic discrimination of *MUS81* c.1292G>A in 362 healthy Brazilian individuals revealed MAF = 0.015; 11 individuals were heterozygous for the variant. Similar MAF was observed in public databases (ABraOM = 0.018; gnomAD Exomes = 0.018; gnomAD Genomes = 0.011, Table S2). In our cohort of 20 unrelated individuals, the *MUS81* c.1292G>A variant had MAF = 0.125, which is 10-fold higher in familial cases compared with healthy Brazilian individuals (Fisher’s exact test *p* = 8.7 × 10^−4^) (Table 1).

Eighty-eight fresh frozen tumor tissues from sporadic BC (*N =* 41) and TC (*N* = 47) were also genotyped (Table S5). Interestingly, three sporadic tumor samples presented a *MUS81* c.1292G>A variant in heterozygosis. The allelic frequency was significantly higher in the familial cohort compared to sporadic tumors (Fisher’s exact test *p* = 6.3 × 10^−3^). Considering the genotype frequencies, the *MUS81* c.1292G>A variant in heterozygosis (GA) was significantly more frequent in the familial cohort compared to healthy controls and sporadic tumors (Fisher’s exact test *p* = 7.1 × 10^−4^ and *p* = 5.3 × 10^−3^, respectively) (Table 1).

### 2.3. MUS81 Protein Expression

**Figure 3:**
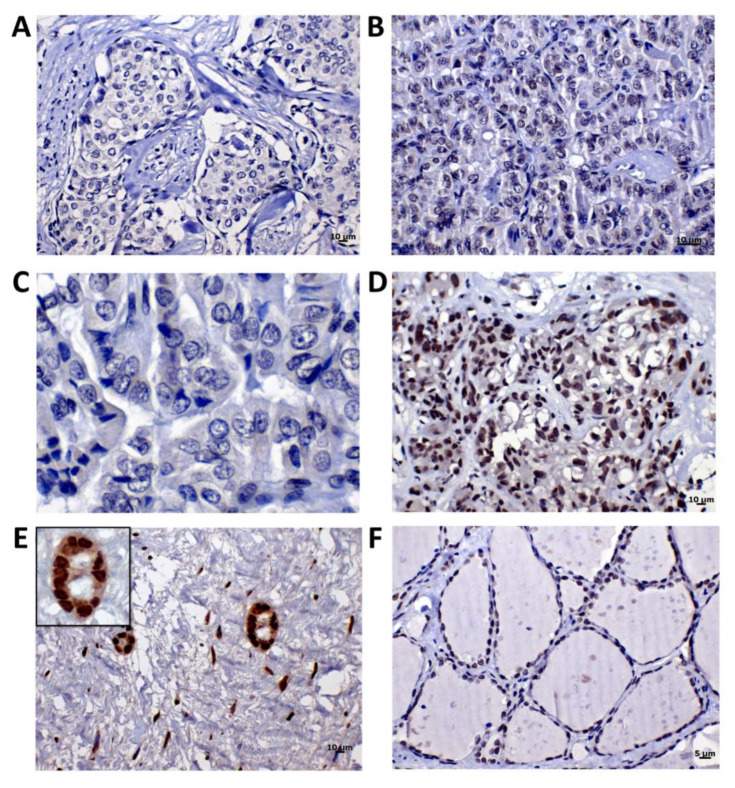
*MUS81* c.1292G>A (p.R431H) variant reduces protein stability. (**A**,**B**) Breast carcinomas with heterozygous *MUS81* variant showed weak protein expression (Score 3). (**C**)Thyroid carcinomas with heterozygous *MUS81* variant showed weak protein immunoreactivity (Score 3). (**D**) Breast carcinoma wild type for p.R431H had moderate to strong MUS81 expression (Score 6). (**E**) Normal mammary gland also wild type for *MUS81* had elevated protein expression (Score 7). (**F**). Normal thyroid tissue wild type for the variant had a moderate protein expression (Score 5).

MUS81 expression is expected to be nuclear [29,30]. We evaluated MUS81 expression in three tumor tissues positive for *MUS81* c.1292G>A (p.R431H) (BC from M4, BC from M5, and TC from M1) and three *MUS81* wild-type tissues (one normal thyroid, one normal breast, and one BC) (Figure 3). Two BCs (Figure 3A,B) and one TC (Figure 3C), from patients M4, M5, and M1, respectively, had weak nuclear protein expression (score 3), while a moderate to strong MUS81 expression (score 6) was detected in the wild-type sporadic BC (Figure 3D). Normal glandular breast tissue had a strong nuclear MUS81 expression (score 7) (Figure 3E), and normal thyroid tissue showed moderate nuclear MUS81 expression (score 5) (Figure 3F). A few stromal cells presented nuclear expression, while the fat tissue was negative for MUS81 immunostaining (Figure 3E). Of note, normal tissue adjacent to the thyroid tumor positive for c.1292G>A presented scatted cells with low MUS81 cytoplasmic expression, as well as a few normal cells with low nuclear MUS81 expression (data not shown). We could not find normal adjacent cells to BC positive for *MUS81* c.1292G>A. These results suggest that MUS81 p.R431H was associated with decreased protein synthesis or stability. The *MUS81* variant status of all formalin-fixed paraffin-embedded (FFPE) samples were confirmed by TaqMan genotyping assay.

### 2.4. MUS81 p.R431H Presents Altered Stability

**Figure 4:**
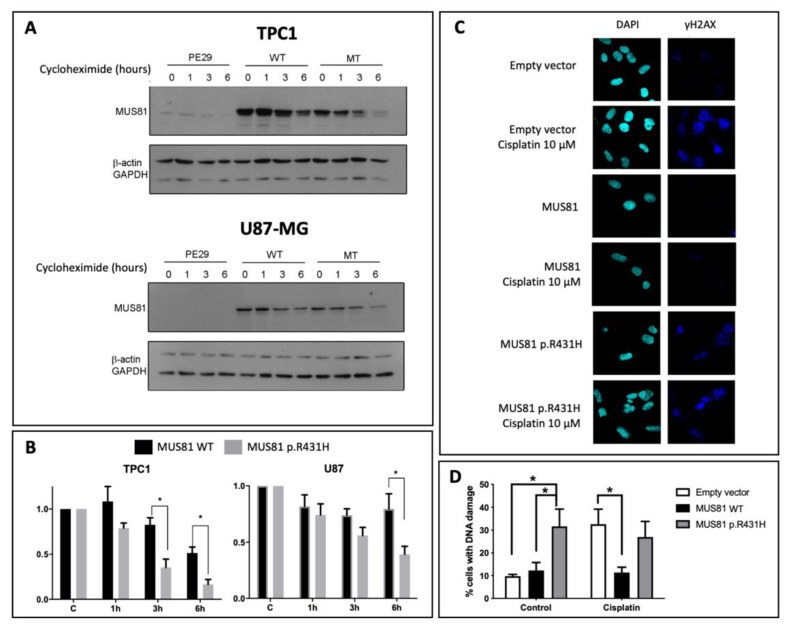
MUS81 c.1292G>A (p.R431H) variant presents reduced stability and reduced DNA damage repair activity. (**A**,**B**) Wild-type (WT) or c.1292G>A (p.R431H) MUS81 were ectopically expressed in TPC1 and U87 cell lines. Protein synthesis was blocked with cycloheximide (CHX) and protein decay after one, three, and six hours was evaluated by Western blot. (**A**) Representative Western; (**B**) quantification of MUS81 levels relative to CHX untreated cells (mean of three independent experiments ± standard error of the mean (SEM)). Two-way ANOVA followed by Dunnett’s post hoc test (WT vs. MT * *p* < 0.05). (**C**). TPC1 cells were transfected with empty vector, MUS81 or MUS81 p.R431H and treated or not with cisplatin for 1 h. Cells were fixed and labeled for phosphorylated Histone H2AX. (**D**). The percentage of yH2AX labeled cells was quantified. Two-way ANOVA followed by Dunnett’s post hoc test (control: Empty vector vs. MUS81 WT *p* > 0.05, Empty vector vs. MUS81 p.R431H * *p* < 0.05; MUS81 WT vs. MUS81 p.R431H * *p* < 0.05; Cisplatin 10 μM: Empty vector vs. MUS81 WT * *p* < 0.05, Empty vector vs. MUS81 p.R431H *p* > 0.05; MUS81 WT vs. MUS81 p.R431H *p* > 0.05).

We investigated whether the MUS81 p.R431H variant protein would present altered stability and/or reduced DNA repair response. Cell lines were transfected with the wild-type *MUS81* (pCMV6-Entry vector containing a *MUS81* [NM_025128] Human complementary DNA (cDNA) ORF (open reading frame), RC203373, OriGene Technologies Inc, Rockville, MD, USA) or mutated *MUS81* c.1292G>A (p.R431H) (constructed by site-directed mutagenesis). Protein stability was measured after inhibition of protein synthesis with cycloheximide. We monitored MUS81 half-life (Figure 4A,B), in cells overexpressing both MUS81 wild type and MUS81 p.R431H. Whereas the protein level of wild type decreased after six hours of synthesis inhibition, a faster decline was observed for MUS81 p.R431H protein, suggesting that this variant enhances protein instability (Figure 4A,B).

Considering that *MUS81* encodes a protein involved in the homologous recombination repair pathway, we investigated the ability of MUS81 p.R431H in the repair of cisplatin-induced DNA damage. Cells overexpressing wild type or MUS81 p.R431H were treated with cisplatin, and rates of DNA damage were measured by phosphorylation of histone H2AX (Figure 4C,D). Overexpression of MUS81 wild type decreases DNA damage caused by cisplatin, while overexpression of MUS81 p.R431H allows greater DNA damage in both untreated and cisplatin-treated conditions (Figure 4D). These findings strongly suggest that MUS81 p.R431H renders cells more susceptible to DNA damage events, which may result in an increased risk of developing cancer.

## 3. Discussion

An association between TC and BC as primary tumors, treated with surgery only, and second primary tumors in young patients, below 40-years-old, was reported as early as 1984, in which a common etiological factor was suggested [31]. A population-based study reported a significantly increased risk of BC and TC for relatives of patients with these tumors [7]. Further studies demonstrated an increased association for the co-occurrence of BC and TC in the same patient, likely due to treatment for the primary site, and in patients having a positive family history of BC and TC [3,4,5,8]. Current genetic evidence for this association is restricted to Cowden syndrome and, in a small proportion, to Li–Fraumeni syndrome, involving pathogenic variants in *PTEN* and *TP53*, respectively [13,32]. However, these variants only explain a small proportion of familial cases. Our findings provide evidence that the *MUS81* c.1292G>A (p.R431H) may explain, at least in part, the familial BC and TC clustering.

We identified variants in cancer-related genes [21,22] that could potentially play a role in familial BC and TC, as well as *MUS81* c.1292G>A penetrance moderator in three patients (M1, M3, and M4) that had both the *MUS81* c.1292G>A and an additional variant in a cancer-related gene. Although patients M3 and M4 had no personal history of polyposis, both patients had the *MUTYH* c.1187G>A (p.G396D) variant. This variant is described as partially defective [33] and is one of the most common variants, together with p.Y179C, associated with bi-allelic *MUTYH* mutation and the recessive form of familial polyposis [34,35]. Of note, the father of patient M4 had colorectal cancer. Patient M4 also had a variant in *ERRC3* (c.2077C>T; p.L693F) that was only reported in the single-nucleotide polymorphism database (dbSNP).

Patient M1 had three variants in cancer-related genes (*PRKAR1A* c.221G>A; *COL7A1* c.7313C>G; and *MTAP* c.634T>C). Inactivating mutations of *PRKAR1A* in TC and, in less frequency, in pancreatic adenocarcinoma patients are associated with Carney Complex syndrome [36]. The *PRKAR1A* variant identified here is described as likely benign in ClinVar because of its higher than expected populational frequency; however, functional assays are necessary to validate this classification. Patient M1 had a family history (maternal and paternal) of pancreatic carcinoma. Variants in *COL7A1* and *MTAP* were not reported in ClinVar; however, variants in these genes are related to squamous cell carcinoma (as part of epidermolysis bullosa spectrum) [37] and sarcoma [38].

Our network analysis revealed that, among 17 cancer-related genes, *MUTYH* and *PRKAR1A* indirectly interact with *MUS81*, via *MSH2* or *CHECK2*, while *ERRC3* directly interacts with *MUS81.* The top significant pathways with related to cancer and *MUS81* were DNA replication, nucleotide excision repair, and lagging strand synthesis. These altered genes can act synergistically and potentially dysregulate the same pathways.

Other recurrently altered genes were detected but, to our knowledge, they were involved in pathways not directly linked to cancer. For instance, the kinesin protein *KIF19* is associated with the transport of membranous organelles and protein complexes in a microtubule- and ATP-dependent manner [39].

Recently, three Brazilian families with hereditary papillary TC investigated by whole-exome sequencing showed pathogenic/likely pathogenic variants in *ANXA3*, *NTN4*, *SERPINA1*, *FKBP10*, *PLEKHG5*, *P2RX5*, and *SAPCD1* [40]. We found variants of unknown significance in *ANXA3* and *FKBP1* in two patients negative for *MUS81* c.1292G>A variant. The authors also reported one family with BC history that harbored *SERPINA1* c.1087G>T (p.G172W) variant. Interestingly, here, we found a pathogenic *SERPINA1* variant (c.1096G>A; p.E366K) in patient W14. *SERPINA1* is a potential candidate associated with the risk of developing familial BC and TC.

Breast and thyroid cancer tissues from patients with germline *MUS81* c.1292G>A (p.R431H) exhibited negative or lower MUS81 expression levels compared with wild-type BC, and normal breast and thyroid tissues. This suggests that MUS81 activity is compromised in tumors of patients positive for p.R431H variant, and conservation of the amino acid is essential for protein function and stability. The MUS81 protein acts in 3′-flap stalled replication fork [41], Holliday structure [42], which prevents chromosomal breaks and deleterious recombination [43,44]. Deregulated expression of *MUS81* was reported in serous ovarian [45] and prostate [46] cancer cells. In hepatocellular [47] and BC cells [48], depletion of MUS81 increased chemosensitivity, highlighting a potential target for cancer treatment. Due to its function in DNA repair, *MUS81* is considered one of the guardians of genome integrity [25].

Consistent with our results in MUS81 protein expression, our functional assays revealed that MUS81 p.R431H is significantly more rapidly degraded than its wild-type counterpart in thyroid and glioblastoma cell models. MUS81 p.R431H also affected the DNA damage response, and its overexpression in cell models was associated with an increase in DNA damage. The analysis of all exons of *MUS81* in the tumor sample from patient M1 confirmed the presence of both germline variants c.1292G>A and c.344C>T. No somatic point mutation was identified. Nonetheless, c.1292G>A might explain the low or negative expression of MUS81 in the tumor, as shown in immunohistochemistry analysis. Of note, *MUS81* was described as a tumor suppressor with a haploinsufficient phenotype [49,50]. Other mechanisms, such as copy number alteration or DNA methylation, could be involved in the *MUS81* regulation. Published data from BC and TC [51,52] showed *MUS81* deletion events and their association with gene down-expression. Moreover, high DNA methylation levels in sites outside the *MUS81* CpG island could also regulate gene expression (Figure S6). Further studies are necessary to address mechanisms involved specifically with the *MUS81* c.1292G>A p.R431H regulation.

Despite *MUS81* c.1292G>A (p.R431H) being reported with MAF = 0.018 in public databases, a significantly higher frequency was detected in our familial group of patients, compared with the healthy Brazilian population (*p* = 8.7 × 10^−4^) and with sporadic BC and TC tissues (*p* = 6.3 × 10^−3^). Recently, a consensual and more stringent MAF threshold was used to facilitate the identification of new pathogenic variants that predispose to rare cancer syndromes. Causal variants classified as pathogenic by the HGMD (Human Gene Mutation Database) and ClinVar databases often have MAF < 0.01% [53]. Nevertheless, a thorough investigation of potential new mutations and/or genes that may display variable penetrance is a valid approach, given that risk alleles may still be hidden in population databases used as controls [54]. For instance, a potentially pathogenic mutation of *TP53* was highly prevalent in population databases, with higher frequencies than previously expected [55]. This finding pointed out the existence of penetrance modifiers even in genes broadly studied in cancer, such as *TP53*. We suggest that *MUS81* c.1292G>A (p.R431H) plays a role in familial breast and thyroid cancer risk. Replication of these findings in larger sample sets is needed to elucidate the penetrance of this variant.

Another important note is that TC is less incident in males compared to females [56,57], and clinical manifestation of hereditary cancer syndromes is often related to cancer diagnosis in the less affected sex, compared to sporadic cancer cases [58]. The brother of the index patient M5 was diagnosed with thyroid cancer at 36 years old, which is considered early onset for sporadic thyroid cancer [56,57].

This study has several limitations. We were unable to confirm the cancer diagnosis in carriers of the *MUS81* variant in relatives of the patient M2 (M2-3 and M2-2) since reports of prophylactic thyroidectomy were provided by the index patient. Although the stringent inclusion criteria strengthened the specificity of our findings, the number of families investigated is small. Furthermore, selecting the most recurrent variant might have introduced bias when comparing the allele frequency between the familial cohort and healthy Brazilian individuals. Other studies using a large cohort of patients and families with a similar phenotype are necessary to validate our findings. We found pathogenic/likely pathogenic variants in cancer-related genes that could contribute to the BC and TC phenotype (e.g., *MUTYH* c.1187G>A and *SERPINA1* c.1096G>A). Unfortunately, the affected relatives of our index patients were treated in other institutions and were not accessible for genetic testing. Future studies would help to clarify whether these variants are associated with BC and TC and/or interact with *MUS81*, contributing to the cancer phenotype. Moreover, investigation of MUS81 protein expression is an important next step to validate the effect of *MUS81* c.1292G>A in a wide cohort of familial and sporadic BC and TC samples.

## 4. Materials and Methods

### 4.1. Patients

In a preliminary survey, we evaluated the clinical data and family history of 130 patients with personal and family history of BC and TC. Then, we established the following patient inclusion criteria: index case with non-medullary TC and/or BC with family cancer history of these cancers AND at least two first- or second-degree relatives with one of these cancers developed before the age of 45 OR the index case plus one first-degree relative affected with one of these cancers with age of cancer onset lower than 40 years old. Exclusion criteria comprised female patients diagnosed with TC and a previous history of BC treated by radiotherapy. Medullary thyroid cancer was excluded due to its association with familial medullary thyroid carcinoma syndrome associated with *RET* mutations (OMIM155240).

Based on these criteria, we selected 20 unrelated patients with a personal and family history of TC and/or BC, which were followed prospectively at the Department of Oncogenetics of the A.C. Camargo Cancer Center, Sao Paulo, Brazil. Subjects provided written informed consent following the Declaration of Helsinki and were advised of the procedures. The study was approved by the institutional Human Research Ethics Committee (FMB-PC-197/2012; CEP1175/08ext).

The medical records reported results of genetic testing for three patients as follows: one patient was tested for *TP53* p.R337H (Brazilian founder mutation for Li–Fraumeni syndrome [59]) and was negative for this variant; one patient tested negative for *TP53* whole-gene, but there was no technical detail; one patient with a putative diagnosis of Cowden syndrome was negative for *PTEN* variants with no further details.

Patients were identified with an alphanumeric character being either F (family with more than one patient assessed by exome sequencing), M (patients positive for *MUS81* c.1292G>A p.R431H), or W (patients *MUS81* wild-type), followed by Hindu-Arabic numerals (1–20). Clinical features and cancer family history of the index cases are detailed in Table S1.

### 4.2. DNA Isolation and Library Construction

Genomic DNA from blood samples (index patients and healthy individuals), saliva (recruited relatives), and frozen tumor tissues (sporadic BC and TC) were extracted using a Qiacube DNA Blood kit (Qiagen, Valencia, CA, USA), Oragene-DNA kit (DNA Genotek, Ottawa, ON, Canada), and Gentra Puregene Tissue Kit (Qiagen, Valencia, CA, USA), respectively. Six microdissected FFPE tissue samples were submitted to deparaffinization, protease digestion, and total DNA extraction using a RecoverAll™ Total Nucleic Acid kit (Ambion, Thermo Fisher Scientific, Waltham, MA, USA). DNA library preparation and whole-exome sequencing were carried out using the Exome Nextera Enrichment kit (Illumina Inc, San Diego, CA, USA) according to the manufacturer’s recommendations and sequenced on Illumina HiSeq 2000 (Illumina Inc, San Diego, CA, USA).

### 4.3. Whole-Exome Sequencing, Bioinformatics Analyses, and Variant Prioritization

Paired-end (PE) raw sequencing data were constructed and sequenced using the Illumina HiSeq 2000 platform (Illumina Inc, San Diego, CA, USA). Read length was 100 bp. PE raw sequencing data had the adaptors trimmed using TrimGalore with default parameters in paired-end mode (https://www.bioinformatics.babraham.ac.uk/), and reads were aligned to the GRCh37/hg19 human reference assembly using BWA-mem version 0.7.15 with default parameters [60]. Duplicate reads (multiple reads that start and end at the same position potentially due to amplification artefacts) were flagged using SAMBAMBA markdup (https://lomereiter.github.io/sambamba/), and base quality scores of the aligned reads were recalibrated using GATK v3.6-0 [61]. Alignment statistics were obtained with Picard (http://picard.sourceforge.net/), SAMtools samtools [62], GATK, and BAMtools (https://github.com/pezmaster31/bamtools). Variants were called with GATK HaplotypeCaller in gVCF mode and genotypes were called for the entire cohort using GATK genotypeGVCFs. Variants were annotated using ANNOVAR [63] and Varseq v2.x (Golden Helix, Inc., Bozeman, MT, USA, www.goldenhelix.com).

We excluded variants as follows: (1) synonymous; (2) observed in more than 2% of the analyzed alleles in GnomAD or AbraOM [64]; (3) with allele fraction <30% (fraction of reads supporting the variant); (4) variants with <10 reads for SNPs and <10 reads for INDELs; (5) variants not shared by all individuals of the same family. This last criterion (5) was adopted for variants detected in the families F1 (W6, W6-1, and W6-2) and F2 (W7 and W7-1).

In silico prediction tools were used to classify the variants and their pathogenicity score. For missense and loss-of-function variants to be predicted as pathogenic in silico, we used CADD with the threshold > 20 [65], while for splice site we used Ada and RF, with the threshold > 0.6 [66]. We also excluded variants classified as benign in ClinVar. We then flagged genes ranked according to high mutation burden due to extensive gene length [24]. From a total of 3855 remaining variants, we focused on variants mapped in cancer-related genes [18,19], recurrently altered genes, and recurrent variants detected in more than one patient/family. A summary of variant prioritization is described in Figure S1. The relationship between the 17 cancer-related genes and MUS81 was illustrated in a PPI network using data from the IID v. 2018-11 [27] (http://ophid.utoronto.ca/iid). We retrieved all direct physical interactions (experimentally identified in human, orthologs from other organisms, and computationally predicted). Final network was visualization using NAViGaTOR version 3.13 [67]. We also used the 17 cancer-related genes and *MUS81*, to perform a pathway enrichment analysis using PathDIP version 4.0.21.2 (Database version 4.0.7.0) [28]. An adjusted *p*-value was obtained using Bonferroni correction at a significance level <0.05.

### 4.4. Data Confirmation

Sanger sequencing analyses were performed to (1) confirm the *MUS81* c.1292G>A variant in five index patients (M1, M2, M3, M4 and M5) and four relatives (M2-1, M2-2, M2-3, and M5-1), (2) confirm the *MUS81* c.1168A>C variant detected in one patient (case M4), and (3) investigate all *MUS81* exons from the tumor of patient M1, which was the only sample available from fresh frozen tissue. Forward and reverse primer sequences are listed in Table S6. In summary, after amplification by conventional PCR, sequencing was performed using the Applied BigDye^®^ Terminator v3.1 Cycle Sequencing Kit protocol (Applied Biosystems, Thermo Fisher Scientific, Waltham, MA, USA). The Prism 3130XL sequencing apparatus (v3.1 Cycle Sequencing, Applied Biosystem, Foster City, CA, USA) was used to run the experiments according to standard protocols. Electropherograms were visualized in CLC Main Workbench (Applied Biosystems, Thermo Fisher Scientific, Waltham, MA, USA).

DNA target genotyping (TaqMan^®^ SNP assay, ID:C_90491711_10, Thermo Fisher Scientific, Waltham, MA, USA) of *MUS81* c.1292G>A was assayed in peripheral blood samples from healthy individuals (*N* = 362) and DNA from fresh frozen sporadic BC (*N* = 41) and TC (*N* = 47) (Table S5). Statistical analyses were performed using Fisher’s exact test in R version 3.4.3 R. We also confirmed the *MUS81* c.1292G>A status in FFPE samples used in the immunoexpression assays (described below), including normal and tumor samples, as well as the tumors of patients M1, M4, and M5. DNA was amplified in the 7900HT Fast Real-Time PCR System (Applied Biosystems, Foster City, CA, USA) with the following cycle conditions: 50 cycles of 95 °C for 10 min, 92 °C for 15 s, and 60 °C for 2 min. The allelic discrimination analysis was performed by Genotyping App in the Thermo Fisher Cloud platform (Applid Biosystems, Foster City, CA, USA).

### 4.5. Immunohistochemistry Analysis (IHC)

Six FFPE tissues were assessed for MUS81 protein immunoexpression including three MUS81 wild-type tissues (one normal thyroid, one normal breast, and one BC) and three MUS81 p.R431H tumor tissues (one BC from patient M4, one BC from patient M5, and one TC from patient M1). Tissue sections (4 mm thick) were dewaxed and hydrated, followed by antigen retrieval using Tris-EDTA (tris(hydroxymethyl)aminomethane-ethylenediaminetetraacetic acid) pH 9.0 solution in a pressure cooker (Pascal^®^, Dakocytomation, Agilent Technologies Inc, Santa Clara, CA, USA). The slides were incubated with methanol containing 0.3% H_2_O_2_ to block the endogenous peroxidase activity. Subsequently, slides were cooled down, and sections were incubated with protein block solution (Protein Block^®^ Dakocytomation, Agilent Technologies Inc, Santa Clara, CA, USA) for 30 min. Sections were incubated with anti-MUS81 monoclonal antibody (Santa Cruz Biotechnology, Dallas, TX, USA) (Table S7) (1:50 dilution) at 4 °C overnight. After rinsing with PBS (phosphate buffered saline), the HRP (horseradish peroxidase)-conjugated secondary antibody was added (Envision, Dakocytomation, Agilent Technologies Inc, Santa Clara, CA, USA) for 1 h at room temperature. All sections were visualized using 3-3′-diaminobenzidine (DAB, Dako Cytomation, Carpinteria, CA, USA) and counterstained with Harris hematoxylin (Millipore, Burlington, MA, USA). The negative control consisted of replacing the primary antibody by mouse immunoglobulin, followed by identical staining procedures. MUS81 expression was classified in scores (0–4) according to the percentage of cells positivity: score 0, negative expression; score 1, <10%; score 2, 11% to 25%; score 3, 26% to 50%; score 4, ≥51%. The intensity of the protein expression was considered as weak (score 1), moderate (score 2), or strong (score 3). The final staining score was calculated adding both scores (range 2–7).

### 4.6. Functional Assessment of MUS81 c.1292G>A

Human thyroid carcinoma (TPC1) cells (BRAF wild type) were obtained from Janete Maria Cerutti (Federal University of São Paulo, Brazil) and subjected to STR analysis [68]. The human glioblastoma carcinoma (U87-MG) cells were purchased from the American Type Culture Collection (ATCC, lot number 5018014). Both cell lines were kept at very low passages and monthly tested to ensure the absence of *Mycoplasma*. TPC1 and U87-MG cell lines were cultured in RPMI (Roswell Park Memorial Institute) 1640 medium (Gibco, Gaithersburg, MD, USA) and Dulbecco’s modified Eagle’s medium with low glucose (Gibco, Gaithersburg, MD, USA), respectively. The medium was supplemented with 10% fetal bovine serum, 100 U/mL penicillin, 100 μg/mL streptomycin, and 250 ng/mL amphotericin B at 37 °C in a humidified 5% CO_2_ atmosphere.

To obtain the *MUS81* c.1292G>A sequence, a pCMV6-Entry vector containing a *MUS81* (NM_025128) Human cDNA ORF (RC203373, OriGene Technologies, Inc, Rockville, MD, USA) was used in site-directed mutagenesis. The c.1292G>A variant was introduced into the wild-type *MUS81* cDNA using sense (5′–CACCCTACGCAGCC**A**CCCCTGGGGAACC–3′) and antisense (5′–GGTTCCCCAGGGG**T**GGCTGCGTAGGGTG–3′) mutated primers (mutated nucleotides are indicated in bold). The reactions were performed using Pfu DNA polymerase (Stratagene, La Jolla, CA, USA) with the following conditions: 95 °C for 1 min, 25 cycles of 95 °C for 30 s, 55 °C for 30 s, 72 °C for 3 min, and final extension of 72 °C for 5 min. The vector containing *MUS81* double-stranded mutant and wild-type DNA was transformed into JM109 Competent Cells (Promega, Fitchburg, WI, USA). Next, the DNA was extracted and purified (NucleoBond^®^ Xtra Midi/Maxi; Macherey Nagel, Bethlehem, PA, USA) using the Wizard Plus SV Miniprep DNA Purification System (Promega, Fitchburg, WI, USA). Successful incorporation of the c.1292G>A variant was confirmed by Sanger sequencing using the BigDye^®^ Terminator v3.1 Cycle Sequencing (Applied Biosystems, Thermo Fisher Scientific, Waltham, MA, USA) and The Prism 3130XL sequencer (Applied Biosystem, Foster City, CA, USA).

The cell lines, U87-MG (8 × 10^4^ cells) and TPC1 (5 × 10^4^ cells) were cultured in plates with 9 cm^2^ surface area. After 24 h, cells were transfected with the constructs (2.5 μg into U87-MG and 3 μg into TPC1) containing the wild-type sequence (here designed as MUS81), the mutant sequence (*MUS81* c.1292G>A; p.R431H), and empty vector using Lipofectamine 2000 (Thermo Fisher Scientific, Waltham, MA, USA) according to the manufacturer’s recommendations. At 24 h post-transfection, cells were incubated for one, three, and six hours with 100 μM cycloheximide (CHX) diluted in DMSO (dimethyl sulfoxide) (Merck & Co, Kenilworth, NJ, USA). The same volume of DMSO was used as a negative control, collected 1 h after incubation. At the time points, cells were taken and processed, and the MUS81 protein was detected by immunoblotting. The experiments were performed in triplicate.

After the drug treatment, U87-MG and TPC1 cells were washed with cold PBS (pH 7.4), and cell extracts were prepared in ice-cold lysis buffer (50 mM Tris, pH 8.0; 150 mM NaCl; 1 mM EDTA; 1% NP-40; 0.5% sodium deoxycholate; 10% of protease) (Roche Diagnostic, Indianapolis, IN, USA) and 10% phosphatase inhibitors (Thermo Fisher Scientific, Waltham, MA, USA). The extracts were centrifuged at 14,000× *g* at 4 °C for 15 min, and the total protein concentration was estimated by using Bradford reagent (Bio-Rad Laboratories, Inc, Philadelphia, PA, USA). A total of 15 μg of protein was mixed with loading buffer (62.5 mM Tris-HCl pH 6.9; 2.5% SDS (Merck & Co, Kenilworth, NJ, USA), 8.7% glycerol (Merck & Co, Kenilworth, NJ, USA), 0.5 mM EDTA, 2.5% β-mercaptoethanol (Merck & Co, Kenilworth, NJ, USA), and bromophenol blue (Merck & Co, Kenilworth, NJ, USA)), denatured by heating at 95 °C for 5 min and subjected to separation in sodium dodecyl sulfate–polyacrylamide gel electrophoresis (SDS-PAGE) gel. The proteins were then blotted onto nitrocellulose membrane and blocked in 5% non-fat milk diluted in a mixture of TBST (tris-buffered saline) (150 mM NaCl; 50 mM Tris pH 7.4; 0.1% Tween^®^ (Sigma Aldrich, Allentown, PA, USA) for one hour at room temperature, followed by incubation with primary antibody (Table S7) overnight in TBST buffer with 5% bovine serum albumin (BSA). The membranes were washed and incubated with peroxidase conjugated-secondary antibody. The bands were visualized using ECL (enhanced chemiluminescent) detection reagents (GE Healthcare, Chicago, IL, USA). Densitometric quantification was performed by ImageJ software (https://imagej.net/ImageJ). The graphics express the protein levels in the relative amount of the membrane stained with Ponceau S solution (Sigma Aldrich-Merck, Germany).

Transfected TPC1 cells (1.5 × 10^5^) were seeded on coverslips in a 24-well culture plate for 24 h. Cells were treated with cisplatin (10 μM) for 1 h, fixed with 4% formaldehyde methanol-free diluted in 1× PBS for 20 min at room temperature. Cells were washed three times with 1× PBS, and the buffer (1× PBS + 0.5% Triton X-100) was added for five minutes, following a blocking step for one hour in 5% BSA in 1× PBS. For the detection of DNA damage, coverslips were incubated with primary antibody Phospho-Histone H2A.X (Ser139) (20E3) Rabbit mAB (Alexa Fluor^®^ 647 Conjugate) (#9720, Cell Signaling, Danvers, MA, USA) and labeled with DAPI (4’,6-diamidino-2-phenylindole) (Molecular Probes, Thermo Fisher Scientific, Whaltam, MA, USA). Coverslips were washed three times with 1× PBS and were mounted onto microscope slides with FluorSave™ (Merck Millipore, Calbiochem, Burlington, MA, USA). The analysis was performed using the Fluoview FV10i (Olympus, Center Valley, PA, USA) counting an average of 700 cells/field in three fields/coverslips. At least 2000 cells were quantified in each condition.

All data generated or analyzed during this study are included in this manuscript and in the Supplementary Materials.

### 4.7. Copy Number Alteration and DNA Methylation from Publicly Available Data

We interrogated publicly available databases to assess copy number alteration and DNA methylation as a potential mechanism involved in *MUS81* gene expression. Invasive ductal breast carcinomas (BC) (*N* = 678) plus adjacent normal tissues (*N* = 85), and papillary thyroid carcinoma (PTC) (*N* = 188) plus adjacent normal tissues (*N* = 21) were obtained from the UCSC Xena (https://xena.ucsc.edu) and cBioPortal (https://www.cbioportal.org) (both accessed on 8 April 2020). Only samples with available copy number alteration, gene expression, and methylation status were selected. Homozygous deletion, single copy deletion, diploid normal copy, low-level copy number amplification, and high-level copy number amplification were identified by the GISTIC algorithm, which combines the frequency and amplitude of an aberration to rates each segment and apply a permutation test to assess the statistical significance. From TCGA (The Cancer Genome Atlas) level 3, we obtained the RNA-seq data, with values log2(*x* + 1) transformed and normalized and methylation (Illumina450K) with beta values ranging from 0–1. Probes that were shown to be cross-reactive and SNP-affected were filtered out. Data analysis and visualization were conducted with statistical and graphical packages available from R. Furthermore, using the aforementioned TCGA BC and PTC and additional 50 PTC paired with the adjacent tissue [51], we investigated methylation probes within and outside CpG island sites of the MUS81 region. We evaluated both copy number alteration and methylation in relation to *MUS81* expression.

## 5. Conclusions

Using the whole-exome sequencing approach, we were able to identify pathogenic and likely pathogenic variants in cancer-related genes and a novel variant mapped to the DNA repair gene *MUS81* associated with familial TC and BC cases. The *MUS81* c.1292G>A variant was confirmed using Sanger sequencing in the index cases and their relatives. This variant was found in low frequency in an independent set of sporadic TC and BC compared to our familial cohort, as well as in healthy individuals. Applying immunohistochemistry and functional analyses, we demonstrated that *MUS81* c.1292G>A disrupts protein stability and affects DNA damage response. According to our in silico PPI analysis, *MUS81* is predicted to interact directly or indirectly with other cancer-related genes, which might also impact the gene penetrance. Based on these data, we suggest that *MUS81* plays a role in the predisposition of familial BC and TC. Other studies are necessary in a large number of cases to confirm this association.

## Figures and Tables

**Table 1:** Allelic and genotypic frequencies of *MUS81* c.1292G>A in the familial cohort, in healthy individuals, and in sporadic breast and thyroid cancers.

Sample Set	Allele					
	G (REF)	Frequency	A (ALT)	Frequency	Total (100%)	*p* *
Families	35	0.875	5	0.125	40	Reference
Healthy	713	0.985	11	0.015	724	8.7 × 10^−4^
Sporadic	173	0.983	3	0.017	176	6.3 × 10^−3^
	**Genotype**					
	GG (WT)	Frequency	GA (HET)	Frequency	Total (100%)	*p* *
Families	15	0.750	5	0.250	20	Reference
Healthy	351	0.970	11	0.030	362	7.1 × 10^−4^
Sporadic	85	0.966	3	0.034	88	5.3 × 10^−3^

REF: reference allele; ALT: altered allele; WT: wild-type locus; HET: heterozygous locus. * Fisher’s exact test.

## Supplementary Materials

The following are available online at https://www.mdpi.com/2072-6694/12/5/1289/s1: Figure S1. Schematic representation of the filtering criteria and variant prioritization used for the whole-exome sequencing data analysis; Figure S2. Sanger sequencing performed in five index patients confirmed the presence of the *MUS81* c.1292G>A variant; Figure S3. Sanger sequencing of *MUS81* performed in thyroid cancer sample from patient M1. Except for exons 3 and 13, no other variant was found; Figure S4. Pedigrees of the index cases positive for the *MUS81* c.1292G>A variant. Heterozygous variants are indicated as GA genotype. Circles and squares represent female and male individuals, respectively. Black arrows indicate the index patients from five families. Diagonal lines indicate deceased members are represented; Figure S5. Allelic discrimination plot from TaqMan Genotyping assay. (A) Eleven of 362 healthy Brazilian individuals were heterozygous for the MUS81 c.1292G>A variant (MAF = 0.015). Five samples did not amplify, and one was undetermined. (B) Three of 88 sporadic thyroid (*N* = 47) and breast (*N* = 41) tumor tissues were heterozygous for the MUS81 c.1292G>A variant (MAF = 0.017). Three samples did not amplify. Blue: homozygous for the wild type allele; green: heterozygous for c.1292G>A allele; yellow: no amplification; light blue: negative control, squares: controls with known genotype; Figure S6. *MUS81* copy number alterations (CNA) and DNA methylation analyses using publicly available data. (A) CNA (left) and DNA methylation (right) profile associated with mRNA expression in 678 invasive ductal breast carcinoma samples from TCGA dataset. Each point indicates a sample. (B) CNA (left) and DNA methylation (right) profile associated with mRNA expression in 188 papillary thyroid carcinoma samples from TCGA dataset. Each point indicates a sample. (C) DNA methylation profile of probes covering *MUS81* in PTC (top) and BC (invasive ductal subtype) samples from TCGA database (mid) and 50 PTC samples from dos Reis MB et al. (2017) (low); Table S1. Characteristics and family history of 20 patients with exome sequencing data analysis; Table S2. Detailed information of variants after data prioritization; Table S3. Number of variants after data prioritization detected in one to five patients; Table S4. Pathway enrichment analysis results from PathDIP using 17 cancer-related genes and *MUS81*. Footnote: Direct interaction: *MUS81*, *CHEK2*, *ERCC3* and *MSH2*; Indirect interaction: *MUS81*, *CHEK2*, *ERCC3*, *MSH2*, *MLH1*, *MUTYH*, *POLE* and *PRKAR1A*; Table S5. Clinical features of patients with papillary thyroid cancer and breast cancer genotyped for *MUS81* c.1292G>A variant; Table S6. Primer sets designed for the *MUS81* gene used for Sanger sequencing; Table S7. Antibodies used in the protein expression analysis.
