# Supplementary material for: Germline Mutation in MUS81 Resulting in Impaired Protein Stability is Associated with Familial Breast and Thyroid Cancer
Source: Cancers (Basel). 2020 May 20;12(5):1289. doi: 10.3390/cancers12051289 (PMC7281423; doi:10.3390/cancers12051289)
Supplement: Supplementary file 1 [file cancers-12-01289-s001.zip › cancers-768495-Supplementary Figures .pdf]

cancers

MDPI

*Article*

# Germline Mutation in *MUS81* Resulting in Impaired Protein Stability Is Associated with Familial Breast and Thyroid Cancer

**Maisa Pinheiro, Fernanda Cristina Sulla Lupinacci, Karina Miranda Santiago, Sandra Aparecida Drigo, Fabio Albuquerque Marchi, Carlos Eduardo Fonseca-Alves, Sonia Cristina da Silva Andrade, Mads Malik Aagaard, Tatiane Ramos Basso, Mariana Bisarro dos Reis, Rolando André Rios Villacis, Martin Roffé, Glaucia Noeli Maroso Hajj, Igor Jurisica, Luiz Paulo Kowalski, Maria Isabel Achatz, and Silvia Regina Rogatto**

**Supplementary Figures**

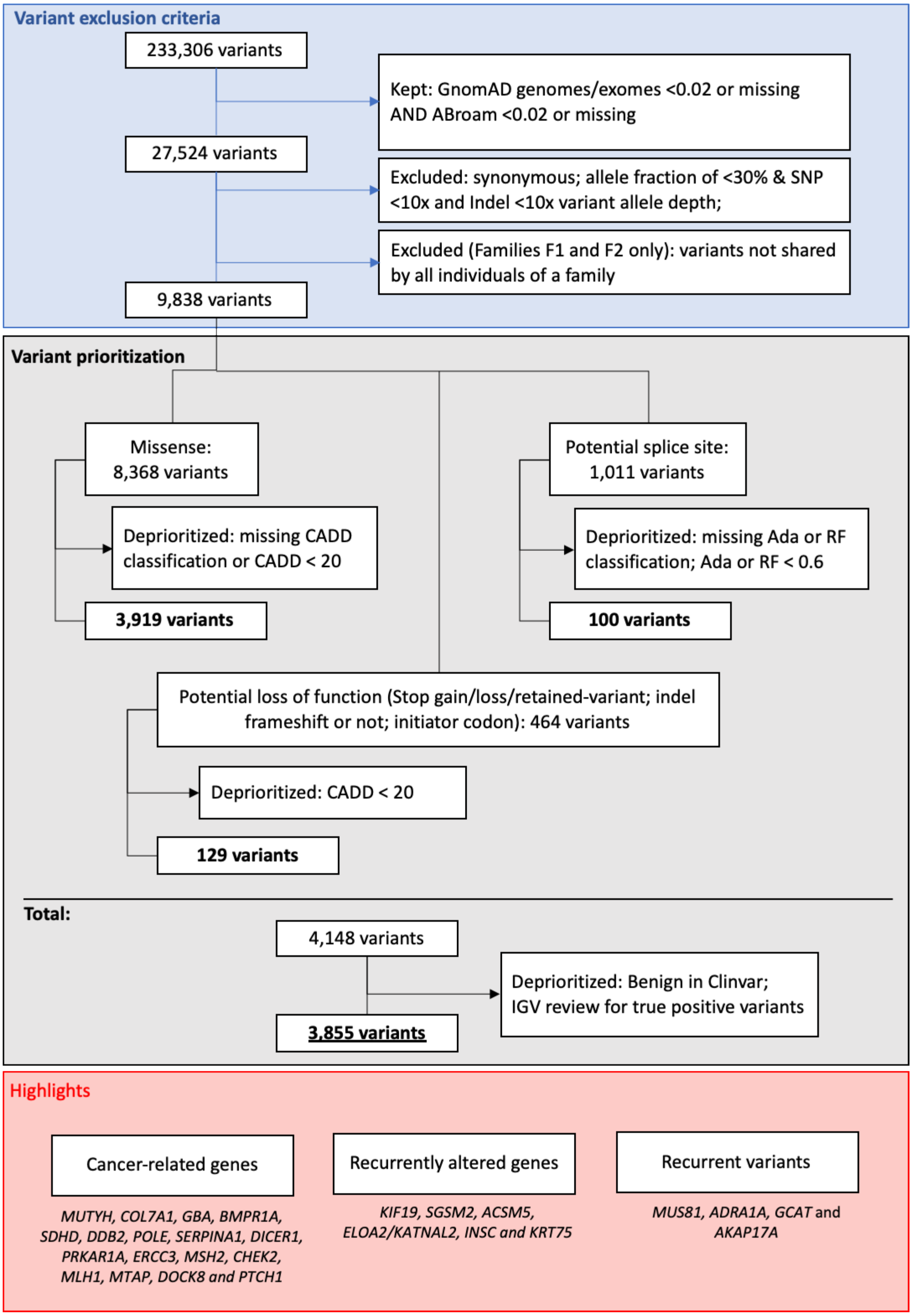


**Figure S1.** Schematic representation of the filtering criteria and variant prioritization used for the whole-exome sequencing data analysis.

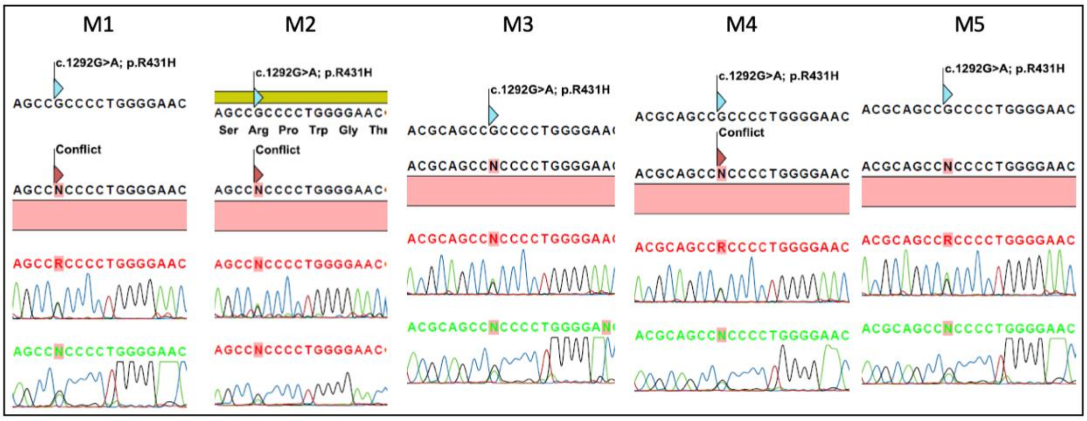


**Figure S2.** Sanger sequencing performed in five index patients confirmed the presence of the *MUS81* c.1292G>A variant.

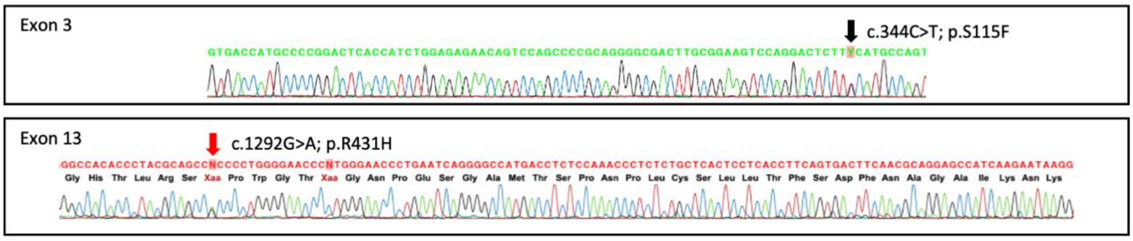


**Figure S3.** Sanger sequencing of *MUS81* performed in thyroid cancer sample from patient M1. Except for exons 3 and 13, no other variant was found.

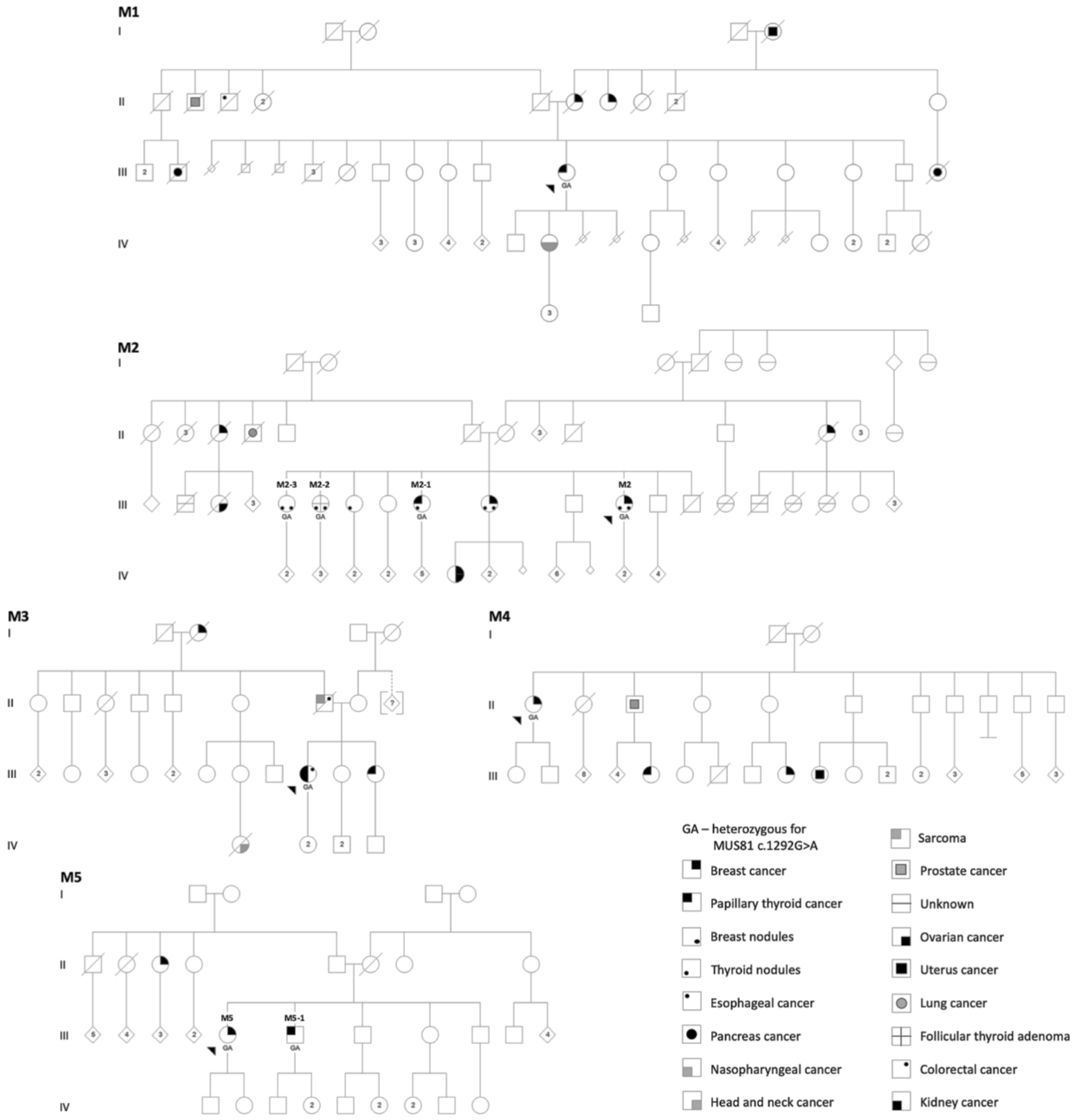


**Figure S4.** Pedigrees of the index cases positive for the *MUS81* c.1292G>A variant. Heterozygous variants are indicated as GA genotype. Circles and squares represent female and male individuals, respectively. Black arrows indicate the index patients from five families. Diagonal lines indicate deceased members are represented.

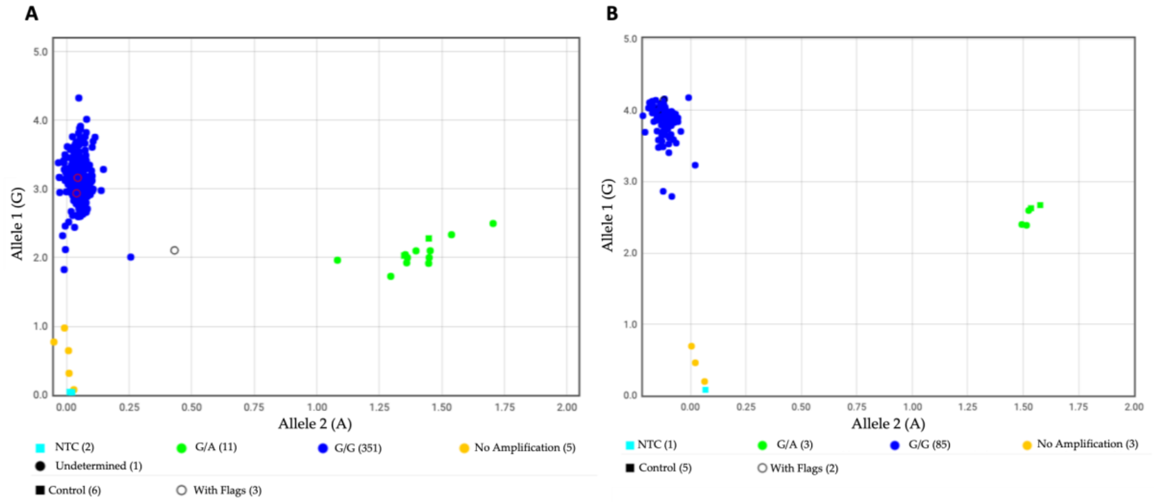


**Figure S5.** Allelic discrimination plot from TaqMan Genotyping assay. (**A**) Eleven of 362 healthy Brazilian individuals were heterozygous for the MUS81 c.1292G>A variant (MAF = 0.015). Five samples did not amplify, and one was undetermined. (**B**) Three of 88 sporadic thyroid (*N* = 47) and breast (*N* = 41) tumor tissues were heterozygous for the MUS81 c.1292G>A variant (MAF = 0.017). Three samples did not amplify. Blue: homozygous for the wild type allele; green: heterozygous for c.1292G > A allele; yellow: no amplification; light blue: negative control, squares: controls with known genotype.

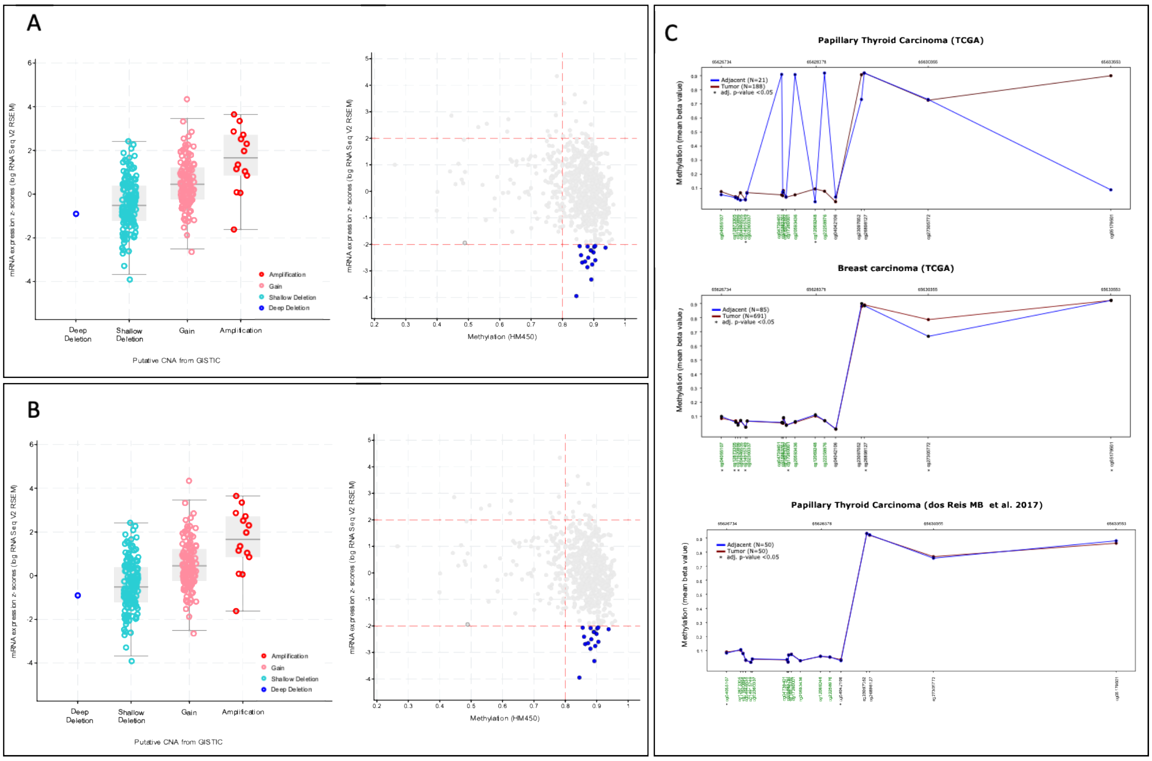


**Figure S6.** *MUS81* copy number alterations (CNA) and DNA methylation analyses using publicly available data. (**A**) CNA (left) and DNA methylation (right) profile associated with mRNA expression in 678 invasive ductal breast carcinoma samples from TCGA dataset. Each point indicates a sample. (**B**) CNA (left) and DNA methylation (right) profile associated with mRNA expression in 188 papillary thyroid carcinoma samples from TCGA dataset. Each point indicates a sample. (**C**) DNA methylation profile of probes covering *MUS81* in PTC (top) and BC (invasive ductal subtype) samples from TCGA database (mid) and 50 PTC samples from dos Reis MB et al. (2017) (low).
